# A Case of Syphilis

**Published:** 1890-08

**Authors:** Mahlon Preston

**Affiliations:** Norristown, Pa.


### A CASE OF SYPHILIS.

Mahlon Preston, M. D., Norristown, Pa.

I cite the following case of venereal infection, with the hope of further fortifying my belief in the possibility of prompt and radical cures of such cases, avoiding the long train of secondary troubles that follow their management by the villainous methods not purely homœopathic.

My earliest knowledge of the present case dates to within ten days of first exposure, at which time no topical or other remedial measures had been used; there is the best reason to suppose no similar infection had ever taken place.

The first inspection disclosed an œdematous prepuce, rather firmly closed in front of the glans, yet permitting a painful and difficult retraction of it, which revealed three large and deep chancres, the size of a split soup-bean. One on either side of the frænum below, the third behind the corona to the right, extending from below to middle way around the glans.

These ulcers were eroding rapidly on the edges, their bottoms appeared dark, granular, and dry.

Induration in and around the sores seemed to threaten a rupture of the parts where the foreskin was retracted. The slightest manipulation was painful to a high degree.

I gave five doses of Merc-sol.$^{6m}$ and a soft pledget of cambric muslin, moistened in milk and water, was closed in beneath the prepuce over the ulcers, to be renewed as required for cleanliness and comfort.

This medicine being permitted to act for three days, examination showed the margin of the foreskin knotted with numbers of minute chancres, similar in quality to those beneath. They had, at the same time, undergone further erosion, so that one-half the glans seemed destroyed from below and behind, leaving simply the urethral channel intact.

This horrid gap had evidently severed the confining hold of the prepuce, since it had retracted beyond the corona and fastened round the remainder of the glans in a vise-like stricture which presented the most forbidding aspect.

The conditions of the case, reviewed with great earnestness at this moment, presented the following points: Patient æt. twenty; slender stature, of good flesh and ruddy, robust appearance, inheriting no dyscrasia; sores deep, hard, of a dark reddish color, rapidly extending, with burning, pricking and itching in them; stiff feeling of penis down to its base; not very copious discharge, but frequent desire thereto, of strong-looking urine containing mucus, giving it a ropy look; tired aching and shooting in the renal region; absence of sexual instinct complete.

This last, in conjunction with the deep and corroding chancres decided for Kali-bichrom. Three doses were given dry and the first signs of improvement became evident twenty-four hours later, and all medicine was withheld while it lasted. During the next fourteen days granulation took place so rapidly that at the end of that period it was nearly complete and the destroyed part had gained its natural shape and prominence, and points of the dermoid covering only wanting, which they speedily received.

Three weeks from first observation of the chancres this had taken place. If the priapism usual in such a state of affairs had prevailed here, the process of separation might have been seriously prevented or retarded. Whereas, the absence of erections permitted and hastened it to the utmost; till the form and substance of the whole organ was within an ace of perfection—now the sexual appetite, which had been from the first so markedly wanting, suddenly asserted itself, and a priapism, causing phymosis, supervened, which at once substituted the new difficulty of danger from sloughing of the head of the penis through strangulation.

Around the frænum the foreskin was œdematous to the size of a goose egg, which extended to the whole length of the penis almost at once, and brought the glans entirely within its cover above, being confined as though tied by a fine cord in three places between it and the pubes; watery, purulent discharge issued from within the foreskin, with itching, pricking, and burning in the neighborhood of the constricted glans. A forcible jerking extended through the whole organ, particularly when patient fell asleep. Merc-corr. first given produced no amelioration, but this painful jerking decided me for Cinnabar, given 5C, three doses, followed by placebo. Improvement came promptly from this latter, giving sleep and comfort to the patient. The first remedy for this symptom had remained without effect, and Cinnab.$^{5c}$ was resorted to, a few doses of which administered at short intervals abated the jerking, the remedy being allowed afterward to exhaust its curative effect. The œdematous condition abated in four days, leaving all the parts in quite a favorable state, and the former chancres entirely cicatrized, an effect hardly to be expected from the amount of tension which seemed to have existed around the healing glans.

Notwithstanding the apparent normal condition now prevailing, a third back-set was encountered in the left eye a few days later. It became injected and swollen, with a very sore spot above the limit of the cornea on the ball. It was described as a protruding and swollen point pricking and wounding the lid when moved, and causing the same feeling in the eyeball itself. Inversion of the lid, indeed, brought this state practically to view, with vascular rays of enlarged and turgid vessels extending over the ball and causing obscuration of a portion of cornea. Very painful, piercing stitches affected the inner canthus with floods of tears, when the eye was opened the slightest, for light could not be borne at all.

Much general smarting and burning affected the eye, and the weight of the lid on the ball seemed almost intolerable. Vision was lost from obscurity of the cornea, yet I could plainly observe the pupil fixed and distorted, being quadrangular in shape.

These appearances and symptoms seemed to denote the probable outbreak of a chancre on the eyeball, and the existence of iritis evidently demanded prompt measures.

This demand, I am sorry to state, was sacrificed in the too hasty giving of Croc-s. for the extreme lachrymation at its first appearance, which was not effective. But a choice was later cast on Clematis E. from a maturer review of the facts present. It has stitches in left eye, inner canthus, as from a pointed instrument, pressure in middle of left eyeball, reddened conjunctiva, profuse lachrymation and dread of light; fear to open the eyes; pain in closing the eyes. So close a reproduction as here existed can cause no surprise at the almost instantaneous changes that come over the case from the administration of Clematis$^{2c}$. To the homœopathist it was as the transmutation from darkness to light, from disease to health. Pain vanished as by magic, and a few days of activity in the absorbents restored normal vision and appearance in and around the eye.

But a mild difficulty was experienced after this. The secondary eruption, which appeared on the face and chest, made but an ephemeral show; it was gradually and steadily removed by Nitric-acid$^{cm}$ given at weekly intervals.

The whole period during which this man remained under treatment was over seven weeks. It exhibited several, if not all the phases of a syphilitic case successively. Six months has now more than elapsed since the last vestige has vanished. Have I a right justly to claim that a radical cure has been made?

